# Temporal patterns of anthrax outbreaks among livestock in Lesotho, 2005-2016

**DOI:** 10.1371/journal.pone.0204758

**Published:** 2018-10-24

**Authors:** Relebohile Juliet Lepheana, James Wabwire Oguttu, Daniel Nenene Qekwana

**Affiliations:** 1 Section Veterinary Public Health, Department of Paraclinical Science, Faculty of Veterinary Sciences, University of Pretoria, Pretoria, South Africa; 2 Department of Agriculture and Animal Health, College of Agriculture and Environmental Sciences, University of South Africa, Florida Science Campus, Johannesburg, South Africa; Spectrum Health, UNITED STATES

## Abstract

**Background:**

Although anthrax is endemic in Lesotho, limited information is available on the patterns of the disease among livestock animals. This study investigated temporal patterns of anthrax outbreaks and cases among livestock animals in Lesotho.

**Methods:**

Secondary data of anthrax outbreaks reported to the Department of Livestock Services between January 2005 and December 2016 was used for this study. Proportions of anthrax outbreaks and cases, and their corresponding 95% confidence interval were calculated and compared across year, season, month and region using the Chi-square or Fisher’s exact test. The autoregression model was used to evaluate annual trends of anthrax outbreaks and cases.

**Results:**

A total of 38 outbreaks were reported in the Lowlands districts of Lesotho. District was significantly (p<0.0001) associated with outbreaks and cases, with the highest proportions of outbreaks (52.6%) and cases (70.2%) reported in Maseru. Significantly (p = 0.0004) higher proportions of anthrax outbreaks (78.9%) and cases (95.1%) were reported in the rainy-hot season compared to the dry-cold season. Five hundred and twenty-six (n = 526) anthrax cases were reported with significantly (p<0.0001) higher proportion of cases (70.3%) in cattle compared to other species. Higher proportion of anthrax cases (35.9%) were reported in 2008 and during the months of February (30.8%) and April (30.2%). There was no significant annual trend in anthrax outbreaks (r = 0.0282; p = 0.6213) and cases (r = 0.0873; p = 0.3512) over the study period.

**Conclusion:**

The burden of anthrax in Lesotho is significantly higher in cattle. Anthrax outbreaks occur only in the lowland districts and follow a seasonal pattern. Therefore, more effort should be targeted at curbing the disease in cattle and the lowlands districts. Furthermore, there should be heightened monitoring of cases in the rainy season to ensure that resultant carcasses are disposed of appropriately to minimise future outbreaks.

## Introduction

Anthrax disease is caused by *Bacillus anthracis*, a gram-positive, aerobic, endospore-forming, and rod shaped bacterium [1]. It is primarily a disease of herbivores, with cattle and sheep being the most affected [2,3]. However, humans can be infected via handling and eating contaminated animal products [1]. Clinical presentation in animals include sudden death with bloody discharge from the natural orifices, bloating, and dyspnea [4].

Outbreaks associated with *B*. *anthracis* have been reported in both developed and developing countries [1,5]. Although, the disease is endemic in South Africa [6], Zimbabwe [7], Namibia [8], and Tanzania [9], it is still under-diagnosed and under-reported [10,11]. Nonetheless, regional and seasonal differences in disease occurrence have been reported [12–14] following a prolonged hot dry spell, preceded by heavy rains [3,9,15]. In Zimbabwe, an increased temporal trend from an annual mean of 3 outbreaks (1967–1971) to 42 outbreaks between 2002 and 2006 was observed [15].

Control of anthrax in endemic areas is implemented by vaccination of susceptible animals and antibiotic therapy may be administered in the early stages of infection. In case of death, infected carcasses can be disposed of by incineration or burial [4]. In Lesotho, all suspected anthrax carcasses must be buried in accordance with the Livestock Industry Proclamation 10 of 1896 [16].

Livestock is the third most important source of income in Lesotho and a major contributor of the country’s gross domestic product (GDP). The agriculture sector contributes 10% of exports, of which, wool and mohair contribute 52%[17]. Therefore, animal mortalities and hindrance of wool and mohair exports associated with anthrax outbreaks have the potential to lower the country’s GDP.

Although anthrax is reported annually in Lesotho, there are no published studies on the patterns of disease occurrence. The present study is based on the hypothesis that anthrax disease is endemic in Lesotho and that outbreaks exhibit no temporal or spatial patterns. Therefore, the aim of this study was to investigate temporal patterns of anthrax outbreaks among livestock in Lesotho between 2005 and 2016. This study being the first on anthrax in Lesotho, serves as a baseline study for future studies of anthrax in Lesotho. Furthermore, by identifying those areas and species with the highest burden this study provides information that can be used to implement a risk-based approach to the control and prevention of anthrax in Lesotho.

## Methods

### Study area

**Fig 1:**
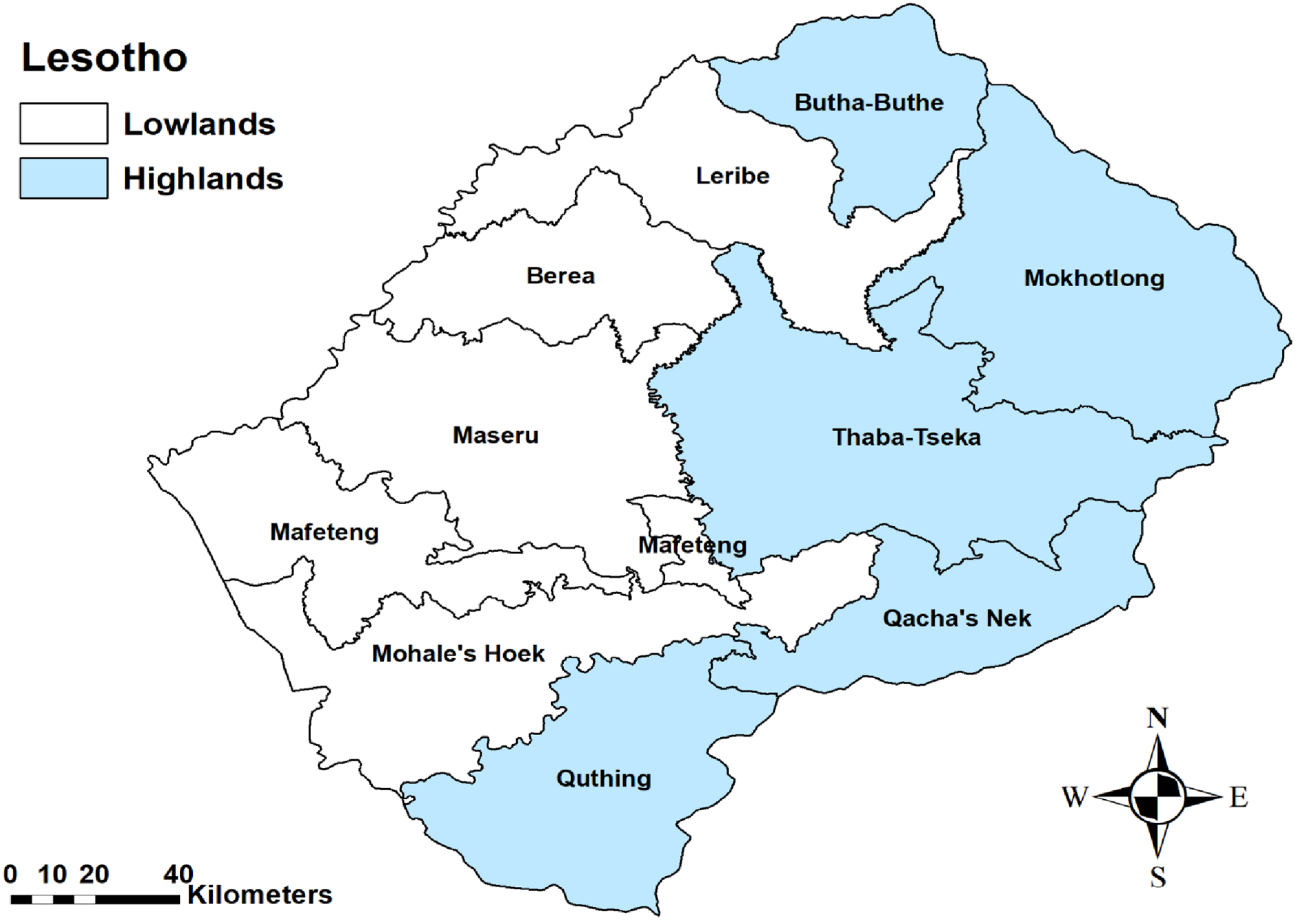
Map showing the ten administrative districts of Lesotho districts and the topographical zones.

Lesotho has 30 355 km^2^ of area landlocked in South Africa, with approximately 540, 133 cattle, 1, 346, 596 sheep, 824, 698 goats and 43, 000 equines (Bureau of statistics, 2015). It is divided into four agro-ecological zones; the lowlands (1,400 to 1,800 m), the Foothills (1,800 to 2,000 m), the Sengu River Valley (1,400 to 1,800 m) and the highlands (2,000 to 3,400 m) above sea level [18]. For this study the country was divided into two topographical zones; the Lowlands (1,400 to 2,000 m) and the highlands (2,000 to 3,400 m) above sea level. In addition, Lesotho is divided into ten districts namely; the highland districts that include Quthing, Qacha’s Nek, Thaba-Tseka, Mokhotlong, Butha-Buthe, and the low land districts that include Leribe Mafeteng, Mohale’s Hoek, Berea and Maseru (Fig 1). The country experiences a rainy-hot season from October to April, with the highest rain falling between December and February, and the dry-cold season which is between May and September [19].

### Data source

Anthrax disease is notifiable under the Lesotho Stock Disease Act; Proclamation 10 of 1896 [16]. Hence, reporting of all suspected cases of anthrax is mandatory. Confirmation of reported cases is based on clinical presentation and microscopic examination of blood smears. Therefore, all cases in the dataset of the epidemiology unit of the Department of Livestock Services (DLS) in Maseru confirmed anthrax cases.

This study used secondary data of all anthrax outbreaks reported to the DLS, the World Animal Health Information System of the World Organization for Animal Health (OIE-WAHIS) and the Animal Resources Information System (ARIS) of the African Union Inter African Bureau for Animal Resources (AU-IBAR) between 2005 and 2016. An outbreak as defined by DLS is an occurrence of one or more cases of anthrax in a location. The following variables were extracted from the data; district, village, date of sample collection and the date of laboratory confirmation, species, number of cases and deaths. Anthrax outbreaks and cases were analyzed at district levels. The project was approved by the Animal Ethics Committee of the Faculty of Veterinary Science, University of Pretoria. Reference number: V087/17".

### Data management and analysis

The data was checked for any inconsistencies including missing values. No inconsistencies were identified in the dataset. All the anthrax outbreaks and cases were aggregated and analysed at district level using SAS 9.4 (SAS Institute Inc., Cary, NC). The Chi-square test was used to assess for association between anthrax outbreaks and season, year, month and district. However, when more than 20% of the cells had expected frequencies <5, Fisher’s exact test was used. Similar tests were done for anthrax cases. The autoregressive model was used to evaluate annual trends in anthrax outbreaks and cases (S1 Appendix). The autoregressive model was chosen because the error terms in time series data are often not independent. Therefore, violating the normal, independent, identically distributed (NII) assumption about residuals required by the OLS regression model. The significance of variables in the model was set at α = 0.05 [20].

## Results

### Anthrax outbreaks

**Fig 2:**
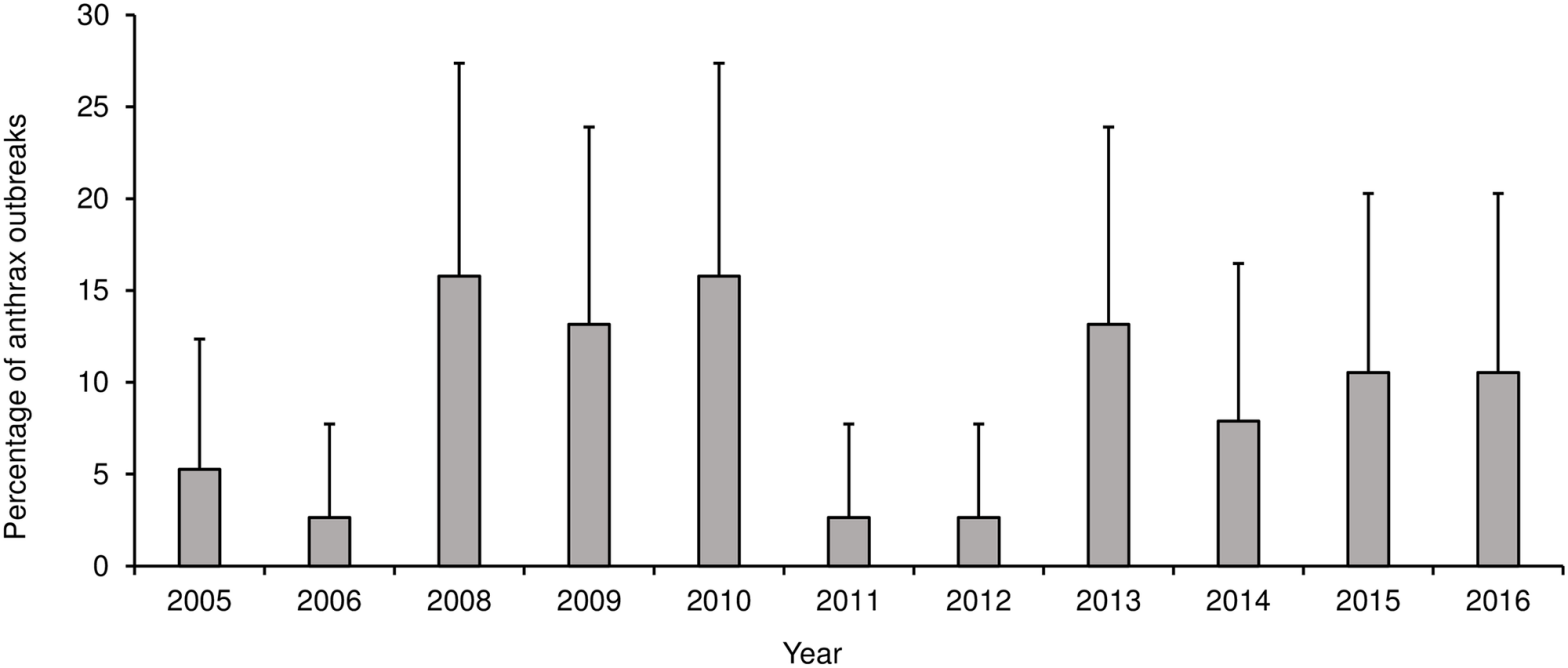
Distribution of anthrax outbreaks in Lesotho between 2005 and 2016. **Notes**: No anthrax outbreaks were reported in the year 2007.

**Fig 3:**
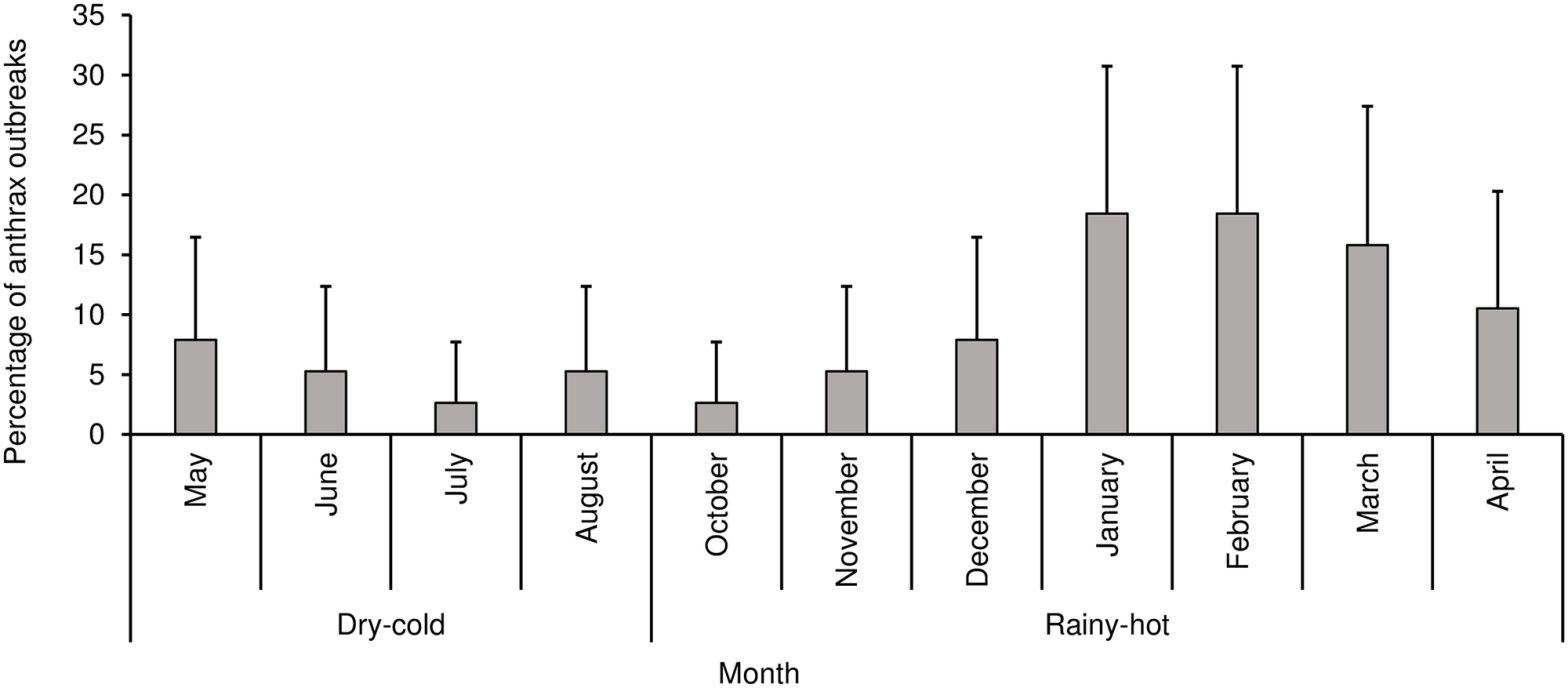
Monthly and seasonal distribution of anthrax outbreaks in Lesotho, between 2005 and 2016. **Notes**: No anthrax outbreaks were reported in the month of September.

No outbreaks were reported from the highlands districts over the study period (2005–2016). There was a significant association between district and number of outbreaks (P<0.0001). Out of 38 outbreaks reported, most outbreaks occurred in Maseru (52.6%; n = 20), followed by Mafeteng (15.8%; n = 6) and Mohale’s Hoek (15.8%; n = 6). The least number of outbreaks were reported in Leribe (5.3%; n = 2). There was no significant annual trend in the number of outbreaks (r = 0.0282; p = 0.6213) (Fig 2). Season was significantly (P = 0.0004) associated with the number of outbreaks, with the rainy-hot season reporting a significantly higher number of outbreaks (79.0%; n = 30) compared to the dry-cold season (Fig 3, Table 1).

**Table 1:** Distribution of anthrax outbreaks in Lesotho by district, year, month and season from 2005–2016.

Variable	Frequency (%)	95% CI
District		
Maseru	20 (52.6)	37.3–67.5
Mafeteng	6 (15.8)	7.4–30.2
Mohale’s Hoek	6 (15.8)	7.4–30.2
Berea	4 (10.5)	4.2–24.1
Leribe	2 (5.3)	1.5–17.3
Year		
2005	2 (5.3)	1.5–17.3
2006	1 (2.6)	0.5–13.5
2008	6 (15.8)	7.4–30.2
2009	5 (13.2)	5.8–27.3
2010	6 (15.8)	7.4–30.2
2011	1 (2.6)	0.5–13.5
2012	1 (2.6)	0.5–13.5
2013	5 (13.2)	5.8–27.3
2014	3 (7.9)	2.8–20.8
2015	4 (10.5)	4.2–24.1
2016	4 (10.5)	4.2–24.1
Month		
January	7 (18.4)	9.2–33.4
February	7 (18.4)	9.2–33.4
March	6 (15.8)	7.4–30.4
April	4 (10.5)	4.2–24.1
May	3 (7.9)	2.7–20.8
June	2 (5.3)	1.5–17.3
July	1 (2.6)	0.5–13.5
August	2 (5.3)	1.5–17.3
October	1 (2.6)	0.5–13.5
November	2 (5.3)	1.5–17.3
December	3 (7.9)	2.7–20.8
Season		
Rainy-hot	30 (78.9)	63.7–88.9
Dry-cold	8 (21.1)	11.1–36.3

**Note**: There were no outbreaks reported in the year 2007 and there were no outbreaks reported in September throughout the study period (2005–2016).

### Anthrax cases

**Fig 4:**
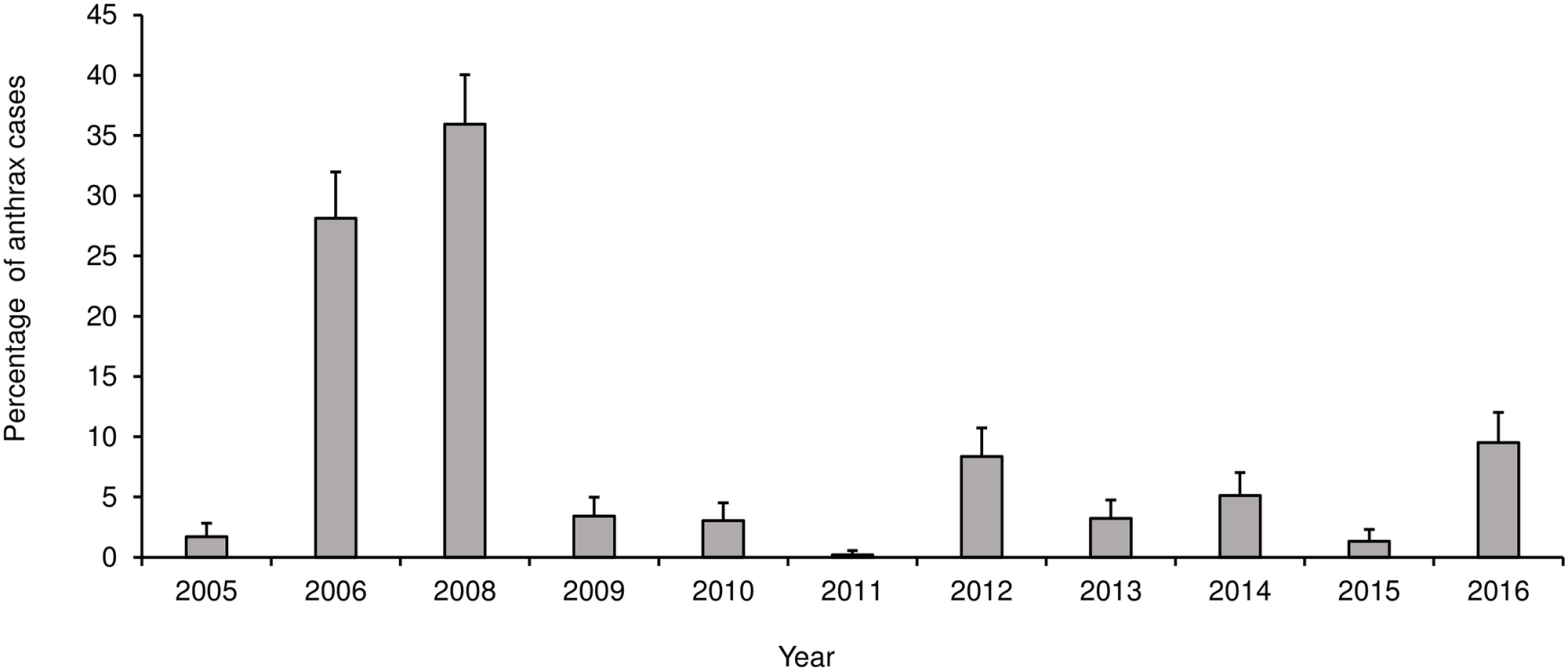
Distribution of anthrax cases in Lesotho between 2005 and 2016. **Notes**: No anthrax cases were reported in the year 2007.

**Fig 5:**
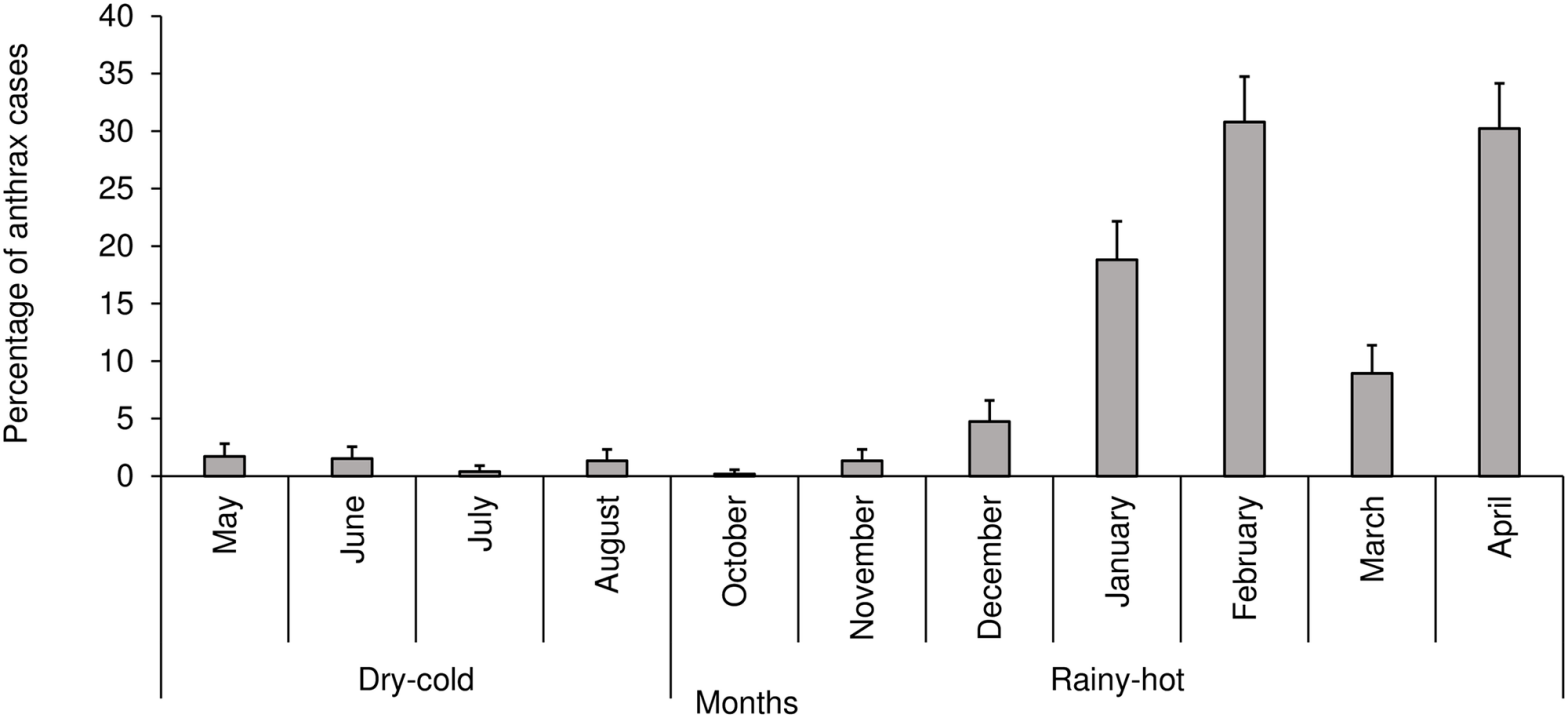
Monthly distribution of anthrax cases in Lesotho between 2005 and 2016. **Notes**: No anthrax cases were reported in the month of September.

**Fig 6:**
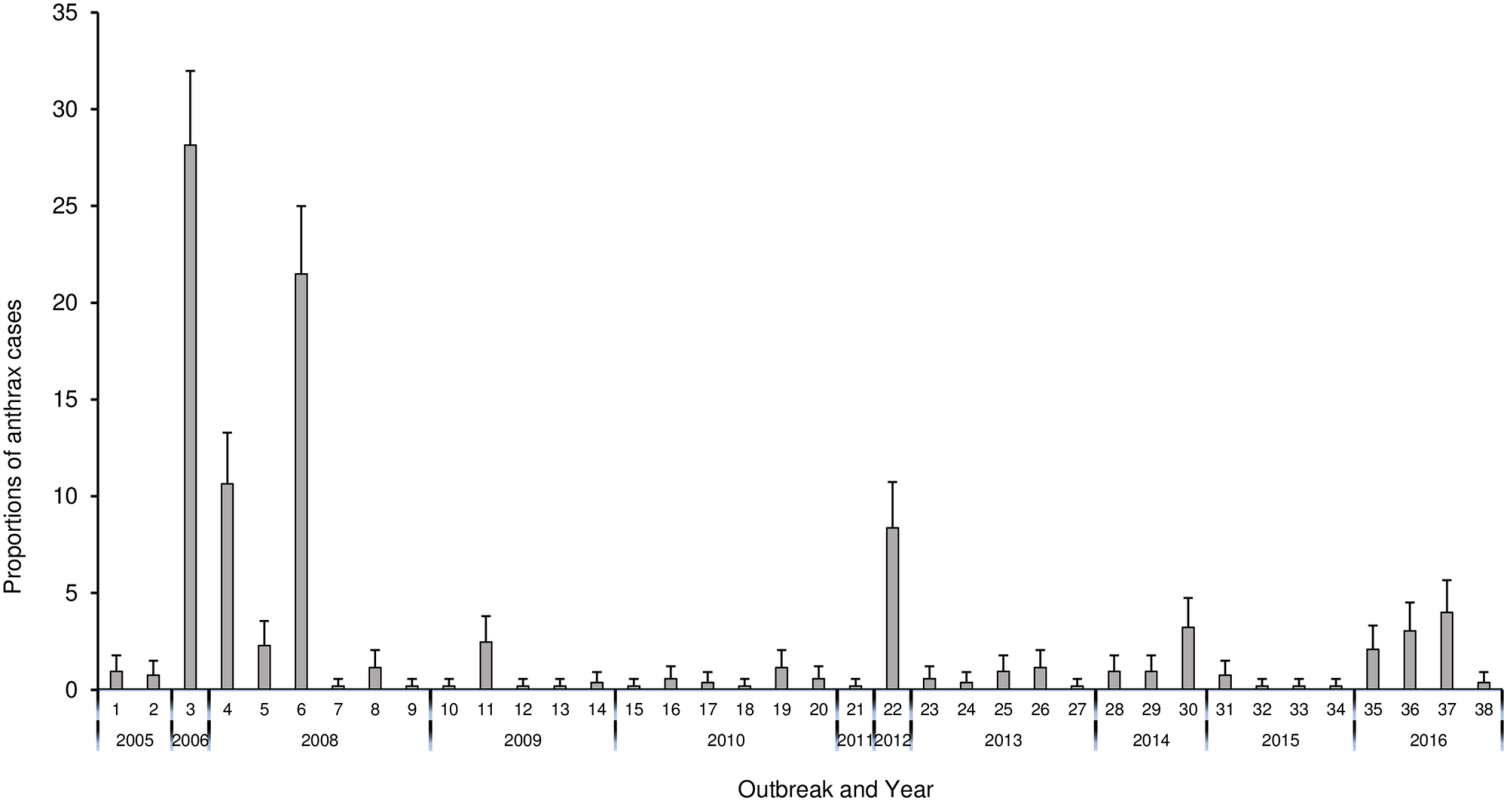
Distribution of anthrax cases per outbreak in Lesotho between 2005–2016. **Notes**: No anthrax cases were reported in the year 2007.

A total of 526 anthrax cases were reported over the study period (2005–2016), and all the cases were from the lowland districts. Thus, the highlands districts did not report any cases. The proportions of cases observed over the years were significantly associated with the type of animal species (P<0.0001), with majority (70.3%; n = 370) of cases reported in cattle. District was highly significantly (P<0.0001) associated with cases, with the majority of cases reported in the Maseru district (70.2%) and very few (0.6%) in Leribe. Year was also significantly (P<0.0001) associated with cases, with the majority of cases reported in 2008 (35.9%) followed by 2006 (28.1%) (Table 2 and Fig 4). There was no significant annual trend in cases (r = 0.0873; p = 0.3512) (Fig 4). Likewise, months were significantly (P<0.0001) associated with the number of cases reported, with February recording the highest number of cases (30.8%) followed by April (30.2%) (Fig 5). High proportion of anthrax cases were reported in the 2006 (28,1%) and 2008 (21.5%) outbreaks, respectively (Fig 6).

**Table 2:** Distribution of anthrax cases in Lesotho by district, year, month and animal species between 2005 and 2016.

Variable	Number (%)	(95%CI)
District		
Berea	51 (9.7)	7.5–12.5
Leribe	3 (0.6)	0.2–1.7
Mafeteng	56 (10.6)	8.3–13.6
Maseru	369 (70.2)	66.1–73.9
Mohale's Hoek	47 (8.9)	6.8–11.7
Year		
2005	9 (1.7)	0.9–3.3
2006	148 (28.1)	24.5–32.1
2008	189 (35.9)	32.0–40.1
2009	18 (3.4)	2.2–5.3
2010	16 (3.0)	1.9–4.9
2011	1 (0.2)	0.0–1.1
2012	44 (8.4)	6.3–11.0
2013	17 (3.2)	2.0–5.1
2014	27 (5.1)	3.6–7.4
2015	7 (1.3)	0.6–2.7
2016	50 (9.5)	7.3–12.3
Month		
January	99 (18.8)	15.7–22.4
February	162 (30.8)	27.0–34.9
March	47 (8.9)	6.8–11.7
April	159 (30.2)	26.5–34.3
May	9 (1.7)	0.9–3.2
June	8 (1.5)	0.8–3.0
July	2 (0.4)	0.1–1.4
August	7 (1.3)	0.6–2.7
October	1 (0.2)	0.0–1.1
November	7 (1.3)	0.6–2.7
December	25 (4.8)	3.2–6.9
Species		
Cattle	370 (70.3)	66.3–74.1
Sheep	86 (16.4)	13.4–19.8
Goats	24 (4.6)	3.1–6.7
Horses	21 (4.0)	2.6–6.0
Pigs	12 (2.3)	1.3–3.9
Donkeys	7 (1.3)	0.6–2.7
Equine	6 (1.1)	0.5–2.5
Season		
Rainy-hot	500 (95.1)	92.9–96.6
Dry-cold	26 (4.9)	3.4–7.1

**Note**: Throughout the study period (2005–2016), no anthrax cases were reported in September month and no cases were reported in 2007

## Discussions

In this study, we assessed the burden and temporal trends of anthrax outbreaks and cases in Lesotho. A total of 38 outbreaks of anthrax were reported in Lesotho between 2005 and 2016. In contrast, Kracalik et al [21] reported 67 anthrax outbreaks in Ghana between 2005 and 2016 and Chikerema et al [15] reported 282 anthrax outbreaks in Zimbabwe between 1967 and 2006. In 2016, livestock populations in Ghana (13 million) and Zimbabwe (8.7 million) were higher than that reported in Lesotho (5.8 million) [22]. Therefore, the differences observed could reflect the burden of the disease in the three countries.

All the outbreaks observed in this study occurred only in the lowland districts. This is consistent with previous reports by Bengis [23], Dragon et al [24] and Chikerema [7] who also observed a high incidence of anthrax outbreaks in the low-lying depressions compared to highlands areas. Successive cycles of flood runoff and the evaporation of water have been known to concentrate the anthrax spores in low-lying areas, which explains the tendency for outbreaks to occur predominantly in the low-laying areas [25]. In addition, Dragon [24] reported high incidences of anthrax outbreaks in areas with an ambient temperatures above 15.5 °C compared to those with ambient temperature <15.5 °C. The authors are of the view this could also explain the high number of outbreaks in the lowlands compared to the highlands of Lesotho. Furthermore, the highlands of Lesotho are cold and experience temperatures as low as -12.5 °C [19]. It is known that adverse environmental conditions like extreme cold are not suitable for survival of the vegetative forms of *B*. *anthracis* cells [24].

The observed association between season and anthrax outbreaks and cases in Lesotho, where proportions of outbreaks and cases were higher in the rainy-hot season compared to the dry-cold season, is consistent with studies done in China [3] and in Tanzania [25]. For example, in Tanzania, it has been reported [25] that a higher number of outbreaks tend to occur in rainy hot season as compared to other seasons. The months of January and February in Lesotho receive the highest rainfall and are often the hottest months [18,26]. It is not surprising that these same months had the highest number of outbreaks and cases reported. Studies have shown that anthrax outbreaks tend to be preceded by either heavy rains and/or prolonged droughts [4,24]. This is confirmed by studies that have reported that anthrax outbreaks usually occur after a prolonged hot dry spell followed by heavy rains [4,9]. In contrast, in Zambia [14], Ghana [21] and Zimbabwe [15], anthrax outbreaks were higher in the hot-dry seasons compared to other seasons.

Studies done in Ghana [21], Kazakhstan [2], Ukraine [12] and China [3] reported higher incidence of anthrax cases in cattle compared to other species. Similarly, we observed higher proportions of anthrax cases in cattle compared to other species. In contrast, in the Serengeti National Park, Tanzania, impalas were the most affected species [9], while in Etosha National Park, Namibia, zebra were primary affected [27]. The reasons for the disparity in species affected could be due to differences in population distribution, feeding habits and types of agricultural systems practiced. For example, in Lesotho, cattle, sheep and goats are usually grazed while equines are housed and fed. Furthermore 70% of cattle in Lesotho are found in the lowland districts that happen to be anthrax endemic areas, while sheep are predominantly kept in the highlands [28]. In addition, our study looked at anthrax in domestic animals while Hampson and Bellan [9,27] reported on anthrax in wild life.

A total of 526 cases of anthrax were reported over an eleven-year period (2005–2016) in this study. This is lower than 2261 cases reported in China over eight years [3] and 851 cases reported in Ghana over eleven years [21]. However, it is possible that the extent of the anthrax situation in Lesotho may be underrated because some cases of anthrax in the most remote areas with poor infrastructure may go unreported.

Although, the highest proportions of anthrax cases were reported in 2008, the 2006 outbreak had the highest number of cases in one outbreak followed by one of the outbreaks in 2008. This could be due to poor vaccination coverage during the previous year.

Lesotho’s annual vaccination reports show that vaccination was not carried out in 2007 contrary to the high number of animals vaccinated at the end of 2006 (S1 Appendix). It is possible that due to the high vaccination coverage in 2006, the immunity of the national herd was high in 2007; which explains why no outbreaks were recorded in 2007. This finding is consistent with the observation that the changes in vaccination policy affects occurrence of outbreaks. For example, Kracalik [29] in Georgia reported an increase in the proportions of anthrax cases in 2008 post annual vaccination policy change in 2007. Similarly, poor anthrax vaccination coverage has also been linked to the high number of cases in Ghana [21].

This study is not without limitations, for example the extent of the anthrax situation in Lesotho may be underrated as mentioned above. This is because some anthrax cases in the most remote areas with poor infrastructure may go unreported. In addition, the authors did not have control over the quality of the data collected, which is a common limitation of retrospective studies. However, several measures can be taken to improve the quality of data collection for future studies. These measures may include standardisation of reporting forms and terminology used to better provide accurate information. In addition, there is a need for training of all stakeholders involved in disease reporting including livestock owners on how to identify outbreaks and cases. Accurate information on population size and geographic location of disease can also be collected for future studies on spatial patterns of disease occurrence. Nonetheless, this study is the first to report on temporal trends and the burden of anthrax outbreaks and cases in Lesotho.

## Conclusion

Anthrax outbreaks occur in the lowlands districts of Lesotho and mainly affect cattle. In view of this, the authors recommend that more effort in terms of surveillance should be directed at the lowlands districts of the country. The outbreaks follow a seasonal pattern with high incidences in the hot-rainy season. Therefore, anthrax control strategies must include vaccination of susceptible animals with more emphasis placed on cattle in the lowlands districts prior to start of the rainy season. Furthermore, the authors recommend heightened monitoring of outbreaks and ensuring of proper disposal of carcasses during the rainy season, to prevent or minimize future outbreaks. It is possible that there are environmental and socio-economic factors influencing the temporal and spatial pattern of anthrax outbreaks in Lesotho. In view of this, the authors recommend that future studies should consider investigating local factors associated with disease outbreaks in the lowlands compared to highlands including soil types.

## Supporting information

S1 Appendix Autoreg. (DOCX)

S2 Appendix Vaccination records. (DOCX)

S3 Appendix Dataset. (XLSX)

## References

[1] Beyer W, Turnbull PCB. Anthrax in animals. Mol Aspects Med. 2009;30: 481–489. 10.1016/j.mam.2009.08.004 19723532

[2] Aikembayev AM, Lukhnova L, Temiraliyeva G, Meka-Mechenko T, Pazylov Y, Zakaryan S, et al Historical Distribution and Molecular Diversity of Bacillus anthracis, Kazakhstan. Emerg Infect Dis. 2010;16: 789–796. 10.3201/eid1605.091427 20409368 PMC2953997

[3] Chen WJ, Lai SJ, Yang Y, Liu K, Li X Lou, Yao HW, et al Mapping the Distribution of Anthrax in Mainland China, 2005–2013. PLoS Negl Trop Dis. 2016;10 10.1371/journal.pntd.0004637 27097318 PMC4838246

[4] World health Organization; Food and Agriculture Organization of the United Nations; World Organisation for Animal Health; Anthrax in humans and animals. Salisburg United Kingdom; 2008.

[5] Barro AS, Fegan M, Moloney B, Porter K, Muller J, Warner S, et al Redefining the Australian Anthrax Belt: Modeling the Ecological Niche and Predicting the Geographic Distribution of Bacillus anthracis. PLoS Negl Trop Dis. 2016;10: e0004689 10.1371/journal.pntd.0004689 27280981 PMC4900651

[6] Smith KL, DeVos V, Bryden H, Price LB, Hugh-Jones ME, Keim P. Bacillus anthracis diversity in Kruger National Park. J Clin Microbiol. American Society for Microbiology; 2000;38: 3780–4. 10.1128/jcm.38.10.3780-3784.2000 PMC87475 11015402

[7] Chikerema SM, Murwira A, Matope G, Pfukenyi DM. Spatial modelling of Bacillus anthracis ecological niche in Zimbabwe. Prev Vet Med. 2013;111: 25–30. 10.1016/j.prevetmed.2013.04.006 23726015

[8] Beyer W, Bellan S, Eberle G, Ganz HH, Getz WM, Haumacher R, et al Distribution and Molecular Evolution of Bacillus anthracis Genotypes in Namibia. PLoS Negl Trop Dis. 1976;6: e1534. 10.1371/journal.pntd.0001534 PMC3295808 22413024

[9] Hampson K, Lembo T, Bessell P, Auty H, Packer C, Halliday J, et al Predictability of anthrax infection in the Serengeti, Tanzania. J Appl Ecol. Blackwell Publishing Ltd; 2011;48: 1333–1344. 10.1111/j.1365-2664.2011.02030.x PMC3272456 22318563

[10] Hugh-Jones M. 1996–97 global anthrax report. J Appl Microbiol. Blackwell Science Ltd; 1999;87: 189–191. 10.1046/j.1365-2672.1999.00867.x 10475945

[11] Sterne M, Nicol J, Lambrechts MC. The effect of large scale active immunization against anthrax. JS AfrVetMedAssoc. 1942;13: 53.

[12] Bezymennyi M, Bagamian KH, Barro A, Skrypnyk A, Skrypnyk V, Blackburn JK. Spatio-temporal patterns of livestock anthrax in Ukraine during the past century (1913–2012). Appl Geogr. 2014;54: 129–138.

[13] Kracalik I, Abdullayev R, Asadov K, Ismayilova R, Baghirova M, Ustun N, et al Changing Patterns of Human Anthrax in Azerbaijan during the Post-Soviet and Preemptive Livestock Vaccination Eras. Zinsstag J, editor. PLoS Negl Trop Dis. Public Library of Science; 2014;8: e2985. 10.1371/journal.pntd.0002985 PMC4102439 25032701

[14] Munang’andu HM, Banda F, Siamudaala VM, Munyeme M, Kasanga CJ, Hamududu B. The effect of seasonal variation on anthrax epidemiology in the upper Zambezi floodplain of western Zambia. J Vet Sci. 2012;13: 293–298. 10.4142/jvs.2012.13.3.293 23000586 PMC3467405

[15] Chikerema SM, Pfukenyi DM, Matope G, Bhebhe E. Temporal and spatial distribution of cattle anthrax outbreaks in Zimbabwe between 1967 and 2006. Trop Anim Health Prod. Springer Netherlands; 2012;44: 63–70. 10.1007/s11250-011-9888-z 21701924

[16] Government of Lesotho. TITLE XXIII LIVESTOCK INDUSTRY PROCLAMATION 10 OF 1896 [Internet]. 1969. https://www.lesothotradeportal.org.ls/kcfinder/upload/files/STOCK_DISEASES.pdf

[17] FAO. Seed Security Assessment—Lesotho 2016 [Internet]. 2016. www.fao.org/3/a-i6086e.pdf

[18] Moeletsi ME, Walker S. Agroclimatological suitability mapping for dryland maize production in Lesotho. Theor Appl Climatol. 2013;114: 227–236. 10.1007/s00704-012-0829-1

[19] Lesotho Meteorological Services. Climate of Lesotho. Minist Energy, Meteorol Water Aff. 2013;

[20] McAllaster L, Douglas L. Basic Usage of SAS or ETS Software to Forecast a Time. The 16th annual NESUG Conference. 2003. https://analytics.ncsu.edu/sesug/2002/ST15.pdf.

[21] Kracalik IT, Kenu E, Ayamdooh EN, Allegye-Cudjoe E, Polkuu PN, Frimpong JA, et al Modeling the environmental suitability of anthrax in Ghana and estimating populations at risk: Implications for vaccination and control. PLoS Negl Trop Dis. 2017;11 10.1371/journal.pntd.0005885 29028799 PMC5656412

[22] OIE World Animal Health Information System OIE World Animal Health Information System. Weekly Animal Disease sevice global report. 2016.

[23] Bengis RG. Anthrax in Free-Ranging Wildlife Fowler’s Zoo and Wild Animal Medicine, Volume 7 2012 pp. 98–107.

[24] Dragon DC, Rennie RP. The ecology of anthrax spores: tough but not invincible. Canadian Veterinary Journal. 1995 pp. 295–301. 7773917 PMC1686874

[25] Turner WC, Imologhome P, Havarua Z, Kaaya GP, Mfune JKE, Mpofu IDT, et al Soil ingestion, nutrition and the seasonality of anthrax in herbivores of etosha national park. Ecosphere. 2013;4: 1–19.

[26] Sumner P. Geomorphic and Climatic Implications of Relict Openwork Block Accumulations Geomorphic and Climatic Implications of Relict Openwork Block Accumulations. Geogr Ann Ser A Phys Geogr. 2004;86 A: 289–302.

[27] Bellan S. Counting Wildlife Carcasses: Anthrax surveillance in Etosha National Park, Namibia Steve Bellan—PhD candidate Department of Environmental Science,. In: Policy. 2011.

[28] Bureau of Statistics. Lesotho Livestock Statistics Report 2013/2014. 2015; www.bos.gov.ls/new%20folder/.../2013_14_livestock_report.pdf

[29] Kracalik I, Malania L, Broladze M, Navdarashvili A, Imnadze P, Ryan SJ, et al Changing livestock vaccination policy alters the epidemiology of human anthrax, Georgia, 2000–2013. Vaccine. Elsevier; 11 2017: 6283–6289. 10.1016/j.vaccine.2017.09.081 28988866

