# Supplementary material for: Temporal patterns of anthrax outbreaks among livestock in Lesotho, 2005-2016
Source: PLoS One. 2018 Oct 24;13(10):e0204758. doi: 10.1371/journal.pone.0204758 (PMC6200195; doi:10.1371/journal.pone.0204758)
Supplement: S1 Appendix — (DOCX) [file pone.0204758.s001.docx]

**OUTBREAKS**

The AUTOREG Procedure

| **Ordinary Least Squares Estimates** | | | |
| --- | --- | --- | --- |
| **SSE** | 125.974178 | **DFE** | 9 |
| **MSE** | 13.99713 | **Root MSE** | 3.74127 |
| **SBC** | 62.8324368 | **AIC** | 62.0366462 |
| **MAE** | 3.07810499 | **AICC** | 63.5366462 |
| **MAPE** | 0.15309346 | **HQC** | 61.5350117 |
| **Durbin-Watson** | 0.1246 | **Regress R-Square** | 0.0282 |
|  |  | **Total R-Square** | 0.0282 |

| **Parameter Estimates** | | | | | | |
| --- | --- | --- | --- | --- | --- | --- |
| **Variable** | **DF** | **Estimate** | **Standard Error** | **t Value** | **Approx Pr > \|t\|** | **Variable Label** |
| **Intercept** | 1 | 2010 | 2.3634 | 850.36 | <.0001 |  |
| **PERCENT** | 1 | 0.1169 | 0.2285 | 0.51 | 0.6213 | Percent of Total Frequency |

**CASES**

The AUTOREG Procedure

| **Ordinary Least Squares Estimates** | | | |
| --- | --- | --- | --- |
| **SSE** | 130.519685 | **DFE** | 10 |
| **MSE** | 13.05197 | **Root MSE** | 3.61275 |
| **SBC** | 67.663747 | **AIC** | 66.6939337 |
| **MAE** | 2.70713006 | **AICC** | 68.027267 |
| **MAPE** | 0.13464452 | **HQC** | 66.3348741 |
| **Durbin-Watson** | 0.3566 | **Regress R-Square** | 0.0873 |
|  |  | **Total R-Square** | 0.0873 |

| **Parameter Estimates** | | | | | | |
| --- | --- | --- | --- | --- | --- | --- |
| **Variable** | **DF** | **Estimate** | **Standard Error** | **t Value** | **Approx Pr > \|t\|** | **Variable Label** |
| **Intercept** | 1 | 2011 | 1.3051 | 1541.07 | <.0001 |  |
| **Cases** | 1 | -0.0175 | 0.0179 | -0.98 | 0.3512 | Cases |
