# Supplementary material for: Temporal patterns of anthrax outbreaks among livestock in Lesotho, 2005-2016
Source: PLoS One. 2018 Oct 24;13(10):e0204758. doi: 10.1371/journal.pone.0204758 (PMC6200195; doi:10.1371/journal.pone.0204758)
Supplement: S2 Appendix — (DOCX) [file pone.0204758.s002.docx]

Lesotho anthrax vaccination records between 2005-2016 (Department of Livestock Services unpublished reports)

| **Year** | **Official vaccination** | **Vaccination in response to an outbreak** |
| --- | --- | --- |
| 2005 | 892'000 | 2'906 |
| 2006 | 0 | 592'000 |
| 2007 | 0 | 0 |
| 2008 | 455'035 | 4'928 |
| 2009 | 18'662 | 405 |
| 2010 | 0 | 10'296 |
| 2011 | 575'435 | 68'262 |
| 2012 | 674 | 258 |
| 2013 | 21'093 | 150 |
| 2014 | 22'035 | 150 |
| 2015 | 456'396 | 0 |
| 2016 | No information | No information |
